# Invasive candidiasis in low birth weight preterm infants: risk factors, clinical course and outcome in a prospective multicenter study of cases and their matched controls

**DOI:** 10.1186/1471-2334-14-327

**Published:** 2014-06-12

**Authors:** Michelle Barton, Karel O’Brien, Joan L Robinson, Dele H Davies, Kim Simpson, Elizabeth Asztalos, Joanne M Langley, Nicole Le Saux, Reg Sauve, Anne Synnes, Ben Tan, Louis de Repentigny, Earl Rubin, Chuck Hui, Lajos Kovacs, Susan E Richardson

**Affiliations:** 1 Division of Microbiology, Hospital for Sick Children, University of Toronto, Room 3654, Atrium, 555 University Ave, Toronto, ON M5G 1X8, Canada; 2 Mount Sinai Hospital, University of Toronto, Ontario, Canada; 3 Stollery Children’s Hospital, Edmonton, AB, Canada; 4 Foothills Hospital, Calgary, AB, Canada; 5 Sunnybrook Health Sciences Centre, University of Toronto, Ontario, Canada; 6 Departments of Pediatrics and Community Health and Epidemiology, Dalhousie University and IWK Health Centre, Halifax, NS, Canada; 7 Children’s Hospital of Eastern Ontario, Ottawa, ON, Canada; 8 Children’s & Women’s Health Centre of BC, Vancouver, BC, Canada; 9 Royal University Hospital, Saskatoon, SK, Canada; 10 CHU Sainte-Justine, Université de Montréal, Montréal, QC, Canada; 11 Montreal Children’s Hospital, Montreal, QC, Canada; 12 Hamilton Health Sciences McMaster University, Hamilton, ON, Canada; 13 Jewish General Hospital, Montreal, QC, Canada

**Keywords:** Invasive candidiasis, Neurodevelopmental outcome, Risk factors, Neonatal, Prematurity

## Abstract

**Background:**

This multicenter prospective study of invasive candidiasis (IC) was carried out to determine the risk factors for, incidence of, clinical and laboratory features, treatment and outcome of IC in infants of birth weight <1250 g.

**Methods:**

Neonates <1250 g with IC and their matched controls (2:1) were followed longitudinally and descriptive analysis was performed. Survivors underwent neurodevelopmental assessment at 18 to 24 months corrected age. Neurodevelopmental impairment (NDI) was defined as blindness, deafness, moderate to severe cerebral palsy, or a score <70 on the Bayley Scales of Infant Development 2nd edition. Multivariable analyses were performed to determine risk factors for IC and predictors of mortality and NDI.

**Results:**

Cumulative incidence rates of IC were 4.2%, 2.2% and 1.5% for birth-weight categories <750 g, <1000 g, <1500 g, respectively. Forty nine infants with IC and 90 controls were enrolled. Necrotizing enterocolitis (NEC) was the only independent risk factor for IC (p = 0.03). CNS candidiasis occurred in 50% of evaluated infants, while congenital candidiasis occurred in 31%. Infants with CNS candidiasis had a higher mortality rate (57%) and incidence of deafness (50%) than the overall cohort of infants with IC. NDI (56% vs. 33%; p = 0.017) and death (45% vs. 7%; p = 0.0001) were more likely in cases than in controls, respectively. IC survivors were more likely to be deaf (28% vs. 7%; p = 0.01). IC independently predicted mortality (p = 0.0004) and NDI (p = 0.018).

**Conclusion:**

IC occurred in 1.5% of VLBW infants. Preceding NEC increased the risk of developing IC. CNS candidiasis is under-investigated and difficult to diagnose, but portends a very poor outcome. Mortality, deafness and NDI were independently significantly increased in infants with IC compared to matched controls.

## Background

The clinical course, including antifungal prophylaxis and treatment of neonatal invasive candidiasis (IC) has been well described in the literature in a number of studies. Although the serious nature of this infection with respect to morbidity and mortality has been described, prospective studies on neurodevelopmental outcome, especially with comparison to a similar pre-term uninfected cohort, have been much less common [1-7]. An early retrospective study of forty six extremely low birth weight (ELBW, <1000 g) infants with *Candida* sepsis or meningoencephalitis reported a significantly higher incidence of short term morbidity (retinopathy of prematurity, chronic lung disease and periventricular leukomalacia), in addition to a higher incidence of adverse neurological outcomes at two years of age compared to ELBW infants without invasive candidiasis [1]. This was followed by a large US prospective study in 2004 of ELBW infants, which reported poor neurodevelopmental outcome as a sequel to neonatal infections in general, including unspecified fungal infections [2]. In the United Kingdom, a national prospective surveillance study of invasive fungal infection (IFI) in very low birth weight infants (VLBW, <1500 g), published in 2006, showed higher mortality in late neonatal and post-neonatal deaths in ELBW infants with IFI compared to those without [3]. The large, landmark US prospective cohort study, published by Benjamin et al. in 2006, reported high rates of morbidity, mortality and neurodevelopmental impairment (NDI) in ELBW infants with IC [4]. In a more recent publication of the same prospective cohort at a later time period, Adams-Chapman et al. showed that ELBW infants with *Candida* sepsis or meningitis had a higher risk of death or NDI than uninfected unmatched ELBW infants obtained from another registry, but not of NDI as an isolated outcome [5]. The rate of death or NDI in the latter two studies was 59 and 73%, respectively [4,5]. The same research group conducted a retrospective analysis of the infants within their database who were managed between 2004 and 2007, and showed that empiric antifungal therapy resulted in improved survival without NDI (as a combined outcome, but not as individual outcomes) in ELBW infants with IC [6]. In a recent retrospective study comparing outcomes in neonatal invasive candidiasis to those in infants with gram negative sepsis, the authors showed that the rate of death or NDI following candidiasis was 48% [7], though this did not differ from the rate in the group with gram negative sepsis. To control for the many factors influencing the manifestation of disease and outcome in preterm neonates, we conducted a prospective, multicenter study of IC in Canadian infants with a birth weight <1250 g, where each case was matched to two controls by gestational age, birth weight, gender and institution of origin. We determined the incidence, risk factors, laboratory features, treatment and mortality of IC, and then followed the surviving cohort longitudinally to assess neurodevelopmental outcome at a corrected age of 18 to 24 months.

## Methods

### Study design

The study was conducted in 13 level III NICUs in nine Canadian cities under the auspices of the Paediatric Investigators Collaborative Network on Infections in Canada (PICNIC) from 2001–2006 (recruitment in the first three years, follow-up extending to 2006), and approved by each Institutional Ethics Review Board:

• Hospital for Sick Children Research Ethics Board

• Mount Sinai Hospital Research Ethics Board

• University of Alberta Heath Research Ethics Board

• Conjoint Health Research Ethics Board, University of Calgary

• Research Ethics Board, Sunnybrook and Women’s College Health Sciences Centre

• Izaak Walton Killam Research Ethics Board

• Children’s Hospital of Eastern Ontario Research Ethics Board

• The University of British Columbia/Children’s and Women’s Health Centre of British Columbia Research Ethics Board

• Biomedical Research Ethics Board, University of Saskatchewan

• Comité d’éthique de la recherché, CHU St-Justine

• Ethics Board of the Montreal Children’s Hospital

• Hamilton Health Sciences/Faculty of Health Sciences Research Ethics Board

• Research Ethics Board of the Jewish General Hospital

No attempt was made to alter the standard of care offered at each center. Routine prophylaxis against IC in high risk infants was not practiced at any center during the course of this study. Definitions used in the study are listed in Table 1.

**Table 1:** Study definitions

**Condition**	**Definition**
Congenital candidiasis	IC within the first 7 days of life
Severe intraventricular hemorrhage (IVH)	Any IVH event ≥ grade 2
Definite CNS candidiasis	Positive CSF or brain culture or yeast forms on histopathology of brain
Probable CNS candidiasis	Positive blood culture for *Candida* plus at least one of:
	CSF WBC count >25 x 10^6^/L in a non-bloody CSF
	CSF protein ≥2.0 g/L
	CSF/serum glucose ratio <0.5
	Head ultrasound compatible with CNS candidiasis
Cerebral palsy (CP)	A non-progressive disorder characterized by abnormal tone in one or more extremities and abnormal control of movement and posture
Moderate to severe CP	Not yet walking at assessment
Severe CP	Unable to sit
Blindness	Visual acuity <20/200 in either eye
Visual impairment	Any visual deficit correctable with glasses
Deafness	Hearing deficit requiring hearing aids or cochlear implants
Hearing impairment	Any hearing deficit not requiring hearing aids or implants
Neurodevelopmental impairment	Any of the following: blindness, deafness, moderate to severe cerebral palsy, or score <70 in PDI or MDI of the BSID-II
*Candida* infection contributing to death	Any of the following: death occurring within 48 hours of a positive culture of *Candida* from a sterile site, death occurring within 48 hours of commencing appropriate antifungal therapy, or documented microbiological or histopathological evidence of *Candida* infection in a normally sterile site at autopsy

In Phase 1 of the study, infants with birth weights <1500 g satisfying inclusion criteria for IC were identified through active laboratory and clinical surveillance. Those that weighed <1250 g were matched with uninfected controls and studied to determine risk factors for acquisition of IC, mortality, and clinical and laboratory features of infection. In Phase 2, the surviving cohort of cases and controls was followed prospectively and assessed for neurodevelopmental outcome at a corrected age of 18 to 24 months.

### Inclusion criteria

Infants with birth weights <1250 g who were less than 90 days of chronological age and had invasive candidiasis, defined as a positive *Candida* culture from a sterile body site (excluding urine), or histopathological, ophthalmologic or autopsy evidence of *Candida* infection. In a previously published report, we described the clinical and mortality outcome in infants with isolated *Candida* UTI identified during the 3 year study recruitment period [8] These infants were not followed beyond discharge to determine long term outcome so are not included in the current study. For the current study, the enrolment date (or day of onset) for each case was the date of the first positive culture from a sterile body site, or the date of diagnosis for cases diagnosed at autopsy, by histopathology or by ophthalmologic examination. For cases of congenital candidiasis, in which *Candida* chorioamnionitis was present, the onset date was considered to be the date of birth.

Two controls without IC were matched to each case by birth weight ± 100 g, gestational age ± 6 days, gender and institution of origin. Controls were required to have survived at least until the chronological age of the respective case at the date of onset of IC. Controls had to have been in the NICU within 12 months of the date of enrolment of the case. Control subjects that developed IC after enrolment were re-enrolled as cases, and new controls were enrolled for the original case.

### Incidence

Data on the incidence of invasive candidiasis were collected for subjects with birth weight <1500 g and stratified into three groups: <750 g, <1000 g and <1500 g. Incidence data were aggregated for each birth weight category from 11/13 NICUs during the first two years of the study, for which we had full surveillance data.

### Risk factor analysis for development of IC and mortality

Matched cases and controls were analysed for the presence of possible risk factors prior to the onset of IC (or the equivalent day of life for the control), such as central venous catheter use, necrotizing enterocolitis, gastrointestinal surgery, parenteral nutrition, duration of intubation, and use of third generation cephalosporins, broad spectrum antibiotics, histamine receptor antagonists, theophylline or systemic steroids.

In a separate unconditional analysis, we explored the role of factors such as gestational age, birth weight, IC, *Candida* CNS infection, necrotizing enterocolitis (NEC) and intraventricular haemorrhage on mortality outcome.

### Outcome measures

For Phase 2, the primary outcome was neurodevelopmental impairment (NDI). A secondary outcome was mortality and/or NDI. Vision, hearing, mental and motor development were assessed at a corrected age of 18 to 24 months by trained staff blinded to case/control status. The Bayley Scales of Infant Development 2nd ed. (BSID-II) [9] were the usual standard for outcome measurement. The Vineland Adaptive Behaviour Scales [10] and Receptive-Expressive Emergent Language Scale (REEL) [11] were administered to infants with developmental delay that was too severe to permit assessment by other methods. For infants that could not be assessed by the Vineland or REEL scales, growth, vision and hearing were measured. The raw scores of the BSID-II generated the mental developmental index (MDI) and psychomotor developmental index (PDI), with a standardized mean value of 100, and a standard deviation (SD) of 15. Scores <2 SD below the mean were considered to be indicative of moderate to severe delay.

### Statistical analysis

It was determined that a sample size of 45 cases was required to allow detection of a significant difference (p <0.05) between cases and controls with 80% power, assuming that those at risk of IC would demonstrate a two-fold higher occurrence of risk factors over the baseline occurrence of 10% in the general VLBW population.

Descriptive statistics, frequency distributions and percentages were calculated for the treatment variables in cases, the outcome variables and other covariates of interest. Univariate conditional logistic regression was conducted to examine potential risk factors for the development of candidiasis as well as to determine whether mortality, NDI or both were associated with IC. Covariates demonstrating p values < 0.2 were selected for entry into the corresponding multivariable conditional logistic regression model. When it was found that the high mortality rate of infants reduced the number of surviving matched pairs for multivariable analysis, an unmatched analysis was performed on the surviving cohort. An unmatched analysis was also conducted to determine factors associated with mortality. Multivariable logistic regression analyses were used to determine the predictors of mortality as well as NDI at 18 to 24 months. Results were reported as odds ratios. Congenital cases and their controls were excluded from the analysis of post-natal risk factors for developing IC, but not from the long-term outcome analysis. Survival curves for cases and controls were estimated using Kaplan Meier analysis and the log-rank test was used to determine whether the difference in survival was significant. SAS (version 9.1; SAS Institute Inc., Cary, NC) was used for the statistical analysis. A p-value of <0.05 was considered statistically significant.

## Results

### Incidence

The two year cumulative incidence rates of IC by birth weight were 1.5% for infants <1500 g (95% CI: 0.09-3.26), 2.2% for infants <1000 g (95% CI: 1.02-3.39), and 4.2% for those <750 g (95% CI: 1.23-7.66).

### Phase 1 demographics

We enrolled 49 infants with IC satisfying inclusion criteria and 90 controls. Only one eligible control per case could be found for eight of the enrolled cases. At enrolment, the median gestational age was 25 weeks (range 23–30). The median chronological age at onset was 14 days (range 0–84) for all cases, but was 20 days (range 9–84) when congenital cases were excluded (Table 2). Male to female ratio was 1.9:1. The mean birth weight was 748 ± 158 g. Cases were not significantly different from controls with respect to demographic and antenatal data, with or without the 15 congenital cases (Table 2).

**Table 2:** Demographic and perinatal features in cases and controls

**Features number (%) or mean ± SD or median (range) unless otherwise stated**	**All cases**	**All controls**	**Significance P value**	**Non-congenital cases**	**Non-congenital controls**	**Significance P value from matched analysis**
	**N = 49**	**N = 90**		**N = 34**	**N = 62**	
Singleton	35 (71)	71 (79)	0.32	26 (77)	50 (81)	0.63
Vaginal delivery	29/49 (59)	50/89 (56)	0.73	16 (47)	31 (51)	0.72
Male	32 (65)	59 (66)	0.97	25 (74)	45 (73)	0.92
White	21/44 (48)	52/87 (60)	0.56	15/31 (48)	33/59 (56)	0.86
Black	7 (16)	13 (15)	1.00	5 (16)	10 (17.0)	0.99
Hispanic	4 (9)	5 (6)	0.92	3 (10)	5 (8)	0.97
Other	12 (27)	17 (19)	0.60	8 (26)	11 (19)	0.63
Mean birth weight (g)	748 ± 159	755 ± 146	0.80	790 ± 162	792 ± 151	0.93
Mean head circumference at birth (cm)	22.9 ± 1.6	23.3 ± 1.9	0.33	23.5 ± 1.6	23.5 ± 1.3	0.93
Mean length at birth (cm)	32.1 ± 2.2	32.9 ± 2.7	0.14	32.5 ± 2.3	33.0 ± 2.4	0.37
Median gestational age (weeks)	25 (23–30)	25.5 (23–30)	0.73	25.5 (23–30)	25.5 (23–30)	0.70
Respiratory distress syndrome	41 (84)	83 (92)	0.35	26 (77)	56 (90)	0.23
Gastrointestinal disorders other than NEC^a^	2 (4)	1 (1)	0.23	2 (6)	1 (2)	0.26
NEC	12 (24)	8 (9)	**0.008**	12 (35)	6 (10)	**0.01**
PDA or other congenital heart disease^b^	37 (76)	63 (70)	0.67	27 (80)	42 (68)	0.25
Intrauterine growth retardation	3 (6)	4 (4)	1.00	3 (9)	4 (6)	1.00
Median age at enrolment (days)	14 (1–84)	14 (0–84)	1.00	20 (9–84)	16 (3–84)	0.89
Median time from admission to death or discharge) (days)	71 (3–492)	89 (1–165)	0.54	91 (3–492)	79 (1–142)	0.09

*Abbreviation*: PDA, patent ductus arteriosus.

^a^All required gastrointestinal surgery and included imperforate anus and jejunal perforation.

^b^36/37 cases and all 63 controls had only PDA.

### Microbiology

*Candida* species were isolated in 47 cases from sterile site specimens [blood alone (n = 30); blood and other sterile sites (n = 14), and sterile sites other than blood (autopsy brain and urine (1), autopsy lung (1), peritoneal fluid (1)). Additionally, 5 of the 49 infants also had placental tissue or amniotic fluid isolates of *Candida.* In the two cases not confirmed by culture, there was confirmatory histopathology from brain tissue obtained at autopsy, and from a resected segment of ileum in an infant who had surgical intervention for NEC with intestinal perforation.

Four of 49 cases (8%) were not detected until autopsy despite all having had at least one blood culture in the week prior to death. These included two cases of CNS candidiasis, one of invasive pulmonary candidiasis and one of *Candida* peritonitis. Twenty-six (53%) cases were due to *C. albicans*, 14 (29%) to *C. parapsilosis,* 4 (8%) to *C. tropicalis*, one to each of *C. guilliermondii*, and *C. glabrata* and one dual infection with *C. tropicalis* (blood) and *C. parapsilosis* (CSF).

### Disease classification

CNS candidiasis occurred in 14 of 28 (50%) infants that had spinal fluid or autopsy evaluation yielding an overall rate of 29% (14/49). Definite CNS candidiasis was present in six cases by CSF culture (n = 3) or autopsy brain culture/histopathology (n = 3), and probable CNS candidiasis in eight cases based on a positive blood culture plus CSF pleocytosis (n = 5) and/or abnormal CSF chemistry (n = 3). Candidemia occurred in 44/49 (90%) cases, of which 14 (32%) had *Candida* spp. identified from at least one other sterile site. *Candida* urinary tract infection occurred in five candidemic infants and preceded one case of CNS candidiasis. *Candida* peritonitis was documented in 3 infants; two had bowel perforation as a consequence of NEC. Congenital candidiasis was diagnosed in 15 of the 49 cases (31%). Six of the 49 cases showed evidence of extensively disseminated disease with spread to more than three organs documented at autopsy.

### Treatment

Forty-five of 49 (92%) infants received antifungal therapy; the remaining four infants died before therapy was instituted. Thirty-two (71%) of the 45 treated infants commenced therapy empirically or within 48 hours of diagnosis (Table 3). Of the 45 treated infants, 25 (56%) received a single antifungal agent; amphotericin B (n = 24) or fluconazole (n = 1). Twenty (44%) received multiple drugs, of which 7 received two (n = 6) or more (n = 1) sequential antifungal drugs and 13 received combination therapy. Combination therapy consisted of amphotericin B plus 5-fluorocytosine (5-FC) in 8 infants (18%), amphotericin B plus fluconazole in 3 infants (7%) and all 3 drugs in 2 infants (4%). Conventional amphotericin B was included in 44/45 (98%) treatment regimens. Ten (23%) cases were later switched to a lipid formulation of amphotericin B (liposomal amphotericin B (n = 4); amphotericin B lipid complex (n = 3) and an unspecified formulation (n = 3)).

**Table 3:** Clinical outcome related to treatment characteristics

**Treatment characteristics**	**Death proportion (%)**	**NDI proportion (%)**
	**[p value**^**a**^**]**	**[p value**^**b**^**]**
Empiric antifungal therapy	3/8 (38) [1.00]	2/5 (40) [0.64]
Antifungal therapy commenced within 48 hours of diagnosis	14/32 (44) [0.92]	8/16 (50) [0.76]
Adequate doses attained within 48 hours of commencing antifungal therapy	17/39 (44) [0.83]	13/20 (65) [0.56]
^c^Single drug therapy	14/25 (56) [0.22]	5/11 (45) [0.72]
^d^Single sequential therapy	0/7 (0) [0.08]	1/6 (17) [0.17]
Received ≤ 14 days of treatment	9/13 (69) [0.11]	2/4 (50) [1.00]

^a^Reference proportion refers to proportion of deaths among infants commencing antifungal treatment =18/45 (40%).

^b^Reference proportion refers to proportion of confirmed NDI among infants who completed therapy and were discharged =15/27 (56%).

^c^Received only 1 drug for entire course of therapy.

^d^Received ≥ 2 drugs over entire course, but only 1 drug administered at any given time.

Conventional amphotericin B was prescribed at starting doses of 0.25-0.49 mg/kg/d (n = 20), 0.5-0.9 mg/kg/d (n = 6) and 1–1.3 mg/kg/d (n = 18) whereas the lipid formulations of amphotericin were dosed at 3 to 6 mg/kg/d in the 10 cases that received this drug. Fluconazole was prescribed at doses ≥ 6.0 mg/kg/d. Dosing of 5-FC was at an average dose of 75 mg/kg/d. Forty-three (96%) of all treated children achieved adequate doses of antifungal therapy within the first 48 hours of commencing treatment.

Of the 14 cases with CNS candidiasis, 8 received multi-drug therapy consisting of amphotericin B plus 5-FC (n = 4), amphotericin plus fluconazole (n = 1), amphotericin followed by fluconazole (n = 2) and all 3 drugs (n = 1). Three of 8 cases died in this group compared to 5/6 in the group that received amphotericin B alone.

### Mortality

**Figure 1:**
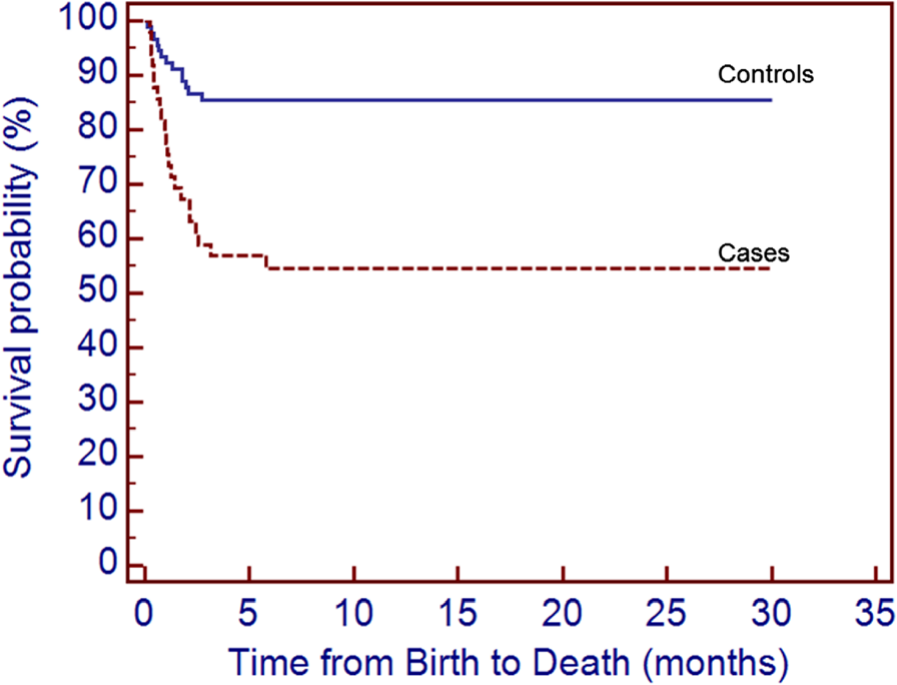
**Survival of very low birth weight preterm infants with IC (followed to 18–24 months corrected age) is significantly less than controls (log-rank test: chi-square 16.46; df =1; HR = 3.74 (95% CI: 1.83- 7.64); p = 0.0001).** Time axis represents chronological age and not corrected age.

Death occurred in 22 (45%) cases versus 13 (14%) matched controls, [OR = 6.00 (2.38 – 15.11); p = 0.0001]. The survival curve was significantly different for cases and controls (p = 0.0001) (Figure 1). Candidiasis was a contributing factor to death in 19 (86%) fatal cases. Death occurred within 3 days of diagnosis in 7 (32%) cases and within 7 days of diagnosis in 10 (45%) cases.

Overall mortality rate among the 45 treated infants was 18/45 (40%) and did not differ significantly for treatment onset prior to or within 48 hours of diagnosis (14/32 (44%); p = 0.82); achievement of adequate dosing within 48 hours of treatment onset (17/39 (44%); p = 0.83); use of single drug therapy (14/25 (56%); p = 0.22); or use of empiric therapy (3/8 (38%); p = 1.00) (Table 3).

### Risk factors

NEC was the only independent risk factor for IC identified (adjusted odds ratio 4.81 [95% CI: 1.14-20.41]; p = 0.03) (Table 4). Factors associated with mortality in the unmatched analysis included IC (p = 0.0002), gestational age (p = 0.012), NEC (p = 0.006), any CNS infection (p = 0.0009), CNS candidiasis (p = 0.013) and either severe intraventricular haemorrhage (IVH) or periventricular leukomalacia (PVL) (p = 0.001). Mortality was not associated with shock requiring inotropic support at enrolment (p = 0.83) or gastrointestinal surgery (p = 0.05). After adjusting for severe IVH or PVL, IC (OR = 3.71 [3.71- 9.11]; p = 0.004), NEC (OR = 6.95 [2.27-21.30]; p = 0.029) and gestational age (OR = 0.60 [0.40-0.88]; p = 0.010) remained independent predictors of mortality.

**Table 4:** Univariate and multivariable matched analysis of potential risk factors preceding enrolment in infants with non-congenital invasive candidiasis

**Factors frequency (%) or median (range) unless otherwise stated**	**Cases**	**Controls**	**Univariate odds ratio**	**Multivariable odds ratio**
	**N = 34**	**N = 62**	**(95% CI) [p value]**	**(95% CI) [p value]**^**a**^
**Central line present**	32 (97)	60 (94)	1.41 (0.19- 10.0)	-
			[0.73]	
**Central line duration (days)**	15.5 (2–37)	14 (2–56)	[0.46]	-
**Not on enteral feeds**	8 (24%)	4 (6%)	**4.28 (1.08-16.54)**	-
			**[0.04]**	
**Duration intravenous lipid (days)**	16 (7–69)	15 (1–35)	[0.09]	1.25 (1.01-1.56)
				[0.0.049]
**Duration intravenous amino acid (days)**	16 (3–70)	16 (6–38)	0.12	-
**NEC preceding IC**^**b**^	12 (35)	6 (10)	**7.04 (1.50-33.30)**	**4.81 (1.14-20.41)**
			**[0.01]**	**[0.03]**
**Bacterial infection including UTI**	17 (50)	19 (31)	2.26 (0.96-5.36)	2.79 (0.57-13.68)
			[0.10]	[0.21]
**Any antifungal therapy prior to IC onset**	13 (35)	10 (17)	2.21 (0.71-6.85)	-
			[0.17]	
**Prior GI surgery**	2 (6)	2 (3)	2.59 (0.03-33.00)	-
			[0.43]	
**H**_**2 **_**blocker**	9 (27)	7 (11)	2.94 (0.96-9.09)	0.67 (0.13-3.46)
			[0.06]	[0.63]
**Steroids**	10 (29)	13 (21)	1.25 (0.34-4.57)	-
			[0.74]	
**Theophylline**	3 (9)	3 (5)	1.56 (0.24-10.56)	
			[0.64]	
**Intubation (median days)**	10 (1–71)	9.5 (1–31)	0.67	-
**Broad spectrum antibiotics**	26 (76)	37 (60)	1.06 (0.97-1.15)	
			0.20	

TPN, total parenteral nutrition.

^a^Only available for factors included in final model; the variable ‘not on enteral feeds’ was not included in the model because this variable overlapped considerably with the variable NEC.

^b^NEC severity was at least stage 2 in 87% of affected cases and 100% of affected controls.

### Phase 2 demographics

Of the 104 surviving infants (27 cases, 77 controls), there were 3 infants who were lost to follow-up, resulting in a total of 25/27 (93%) surviving cases and 76/77 (99%) surviving controls that had long-term follow-up. At follow-up assessment, cases were not significantly different from controls in demographic characteristics (Table 5).

**Table 5:** Demographic and neurodevelopmental characteristics of survivors at follow-up

**Characteristics frequency (%) or mean** **±** **SD, where relevant**	**Cases**	**Controls**	**Univariate odds ratios**^**a**^	**P value**
	**N = 25**	**N = 76**	**[95% CI]**	
Corrected age at final follow-up (months)	20.4 ± 2.9	20.5 ± 3.5		0.94
Males	19 (76)	51 (67)	1.55 [0.55-4.37]	0.46
Gestational age (weeks)	25.4 ± 1.2	25.3 ± 1.5		0.77
Birth weight (g)	763 ± 151	785 ± 150		0.53
Any hearing deficit^b^	7 (28)	13 (18)	1.82 [0.63-5.26]	0.26
Deafness	7 (28)	5 (7)	**5.37 [1.52-18.91]**	**0.01**
Visual impairment	6 (24)	20 (26)	0.88 [0.31-4.53]	1.00
Blindness	1 (4)	1 (3)	3.08 [0.04- 248.49]	0.44
Severe CP	2 (8)	3 (4)	2.12 [0.33-13.45]	0.60
Moderate or severe CP	6 (24)	6 (8)	3.63 [0.86-15.33]	0.067
Mean PDI^c^	80.5 ± 17.9	82.0 ± 16.7		0.74
[Median (range)]	[83.5 (49–107)]	[84.5 (49–107)]		[0.71]
Mean MDI^c^	76.7 ± 20.5	82.1 ± 19.0		0.89
[Median (range)]	[72 (49–111)]	[81 (49–133)]		[0.23]
BSID-II <2 SD	13/23 (57)	23/70 (33)	**2.66 [1.01-6.96]**	**0.044**
NDI^d^	15/25 (60)	25/76 (33)	**3.06 [1.20-7.77]**	**0.017**

^a^Odds ratios were determined analysing the surviving cohort as unmatched cases and controls to maximize sample size and allow multivariable analysis of NDI.

^b^Deafness or hearing impairment.

^c^MDI and PDI Scores that were <50 were assigned a value of 49 to allow determination of means and medians.

^d^Restricting analysis to only matched pairs, using conditional logistic regression, cases were still more likely to develop NDI than their matched controls (10/19 (53%) cases versus 11/38 (29%) controls; **OR = 4.81 [1.32-17.56]**.

### Developmental assessment

Twenty three of 25 (92%) surviving cases and 70/76 (92%) surviving controls had neurodevelopmental assessments completed. Formal testing was not possible in another 2 cases and 3 controls because they had severe developmental delay. Three controls were lost to follow-up.

### Neurodevelopmental morbidity

NDI occurred in 15 (56%) cases versus 25 (33%) controls (p = 0.017; Table 4) and was not significantly different for treatment variables examined (p > 0.05; Table 3). When the analysis was restricted to only those surviving cases in which the matched controls survived, we found that cases were still more likely than matched controls to develop NDI (10/19 (53%) cases versus 11/38 (29%) controls; OR = 4.81 [1.32-17.56]).

Of the four survivors of CNS candidiasis that were available for follow-up, two (50%) had NDI. Deafness occurred more often in IC cases than controls (p = 0.01) but among the seven cases with documented deafness, only two (28.6%) had documentation of CNS candidiasis. Of note, aminoglycoside use did not differ between cases [5/7 (71%)] and controls [3/4 (75%); p = 0.06] with deafness. Although mean and median BSID-II scores were not significantly different between cases and controls, more cases than controls had BSID-II scores < 2 SD below the mean [13/23(57%) vs. 23/70 (33%); p = 0.044], indicating moderate to severe delay (Table 5).

### Factors predicting neurodevelopmental impairment

Gestational age (p = 0.015) and IC (p = 0.017) were significantly associated with NDI, while CNS candidiasis (p = 0.62), gender (p = 0.75), oxygen requirement at 36 days (p = 0.08), head ultrasound abnormality (p = 0.10), severe IVH/PVL (p = 0.20), ROP > stage 2 (p = 0.62) and any CNS infection (p = 0.18) were not, in the univariate analysis. After adjusting for any CNS infection and oxygen requirement at 36 days, IC (OR =3.48 [1.24-9.75]; p = 0.018) and gestational age (OR = 0.57 [0.38-0.87]; p = 0.008) remained predictors of NDI. Switching any CNS infection for severe IVH/PVL in the model resulted in similar results, with IC (p = 0.19) and gestation (p = 0.007) as the only independent predictors. Death or NDI was more likely in infants with IC than their matched controls (OR = 5.03 [2.01-12.75]; p = 0.001).

## Discussion and conclusions

Our findings demonstrate that invasive candidiasis has a significant adverse impact on neurodevelopmental outcome and mortality in an infant population that is already seriously at risk. Infants with IC showed a three-fold higher mortality rate over the control population (45% vs. 14%) and a combined death or NDI rate of 76% compared to controls (42%, p = 0.001). The 45% mortality rate in our study was higher than the 19 to 32% reported elsewhere [4,12,13], but the rate of death and/or NDI was similar to that reported by Benjamin et al. [4] and Adams-Chapman et al. [5] using similar definitions. NDI alone occurred more often in cases than in unmatched controls and remained significant, with an OR of 4.81 when only matched pairs were analysed. Unique to our study was the finding that infants with IC had a significantly higher risk of NDI than uninfected control infants, not just a higher risk of the combined outcome of NDI and mortality. This finding establishes the role of IC in causing impaired neurological development in low birth weight preterm infants, quite apart from its effect on mortality. We demonstrated that IC, along with gestational age and NEC, predicted mortality.

The frequency of deafness was higher in cases compared to controls (28% vs. 7%). Deafness was present in 50% of meningitis survivors with follow-up, despite similar use of aminoglycosides in both groups. The association of hearing impairment with IC was also documented by Benjamin et al. [4] as well as by de Haan et al. [7]. However, in contrast to our study, Benjamin’s study did not show hearing impairment in the 15 *Candida* meningitis survivors, and de Haan’s study did not report on the relationship of hearing impairment to meningitis.

Variables related to the drug, dose, timing and duration of treatment did not affect the mortality rate in IC cases. Prompt treatment and appropriate dosing for neonatal IC are felt to be important management strategies to rapidly control the progress of the infection and to improve clinical outcome. Although we did not observe an improved outcome in infants who were treated more quickly or dosed at therapeutic levels more rapidly than others, we did observe that only 71% of infants began antifungal therapy within 48 hours of the diagnosis being made. A contributing factor was the delay in communicating the positive reports of histopathology and culture of the placentas from birth centers to referral centers.

The overall rate of definite or probable CNS candidiasis documented in our study was 29%. However, when only the infants who had lumbar punctures were considered, CNS candidiasis occurred in 50%. Although our overall rate of CNS candidiasis was similar to that reported in the retrospective review by Fernandez et al. (30%) [14], it was considerably higher than the 5% reported in the U.S cohort study [4]. Variation in rates of lumbar puncture and definitions of CNS candidiasis may partly explain this discrepancy. Most importantly, in our study, children with CNS candidiasis had high rates of death (57%), NDI (50%) and deafness (50%). However, as spinal taps were not performed on 43% of infants with IC, the finding that NDI occurred at a similar rate in IC survivors with or without documented CNS infection, suggests that there may have been missed cases of CNS candidiasis among those that were not completely assessed.

We and others [14,15] have found that CSF culture is insensitive for the diagnosis of CNS candidiasis; one of our cases demonstrated evidence of florid CNS infection at autopsy despite a negative CSF culture. This may relate to the fact that CNS candidiasis can manifest as a multi-focal parenchymal infection rather than as meningitis. Our results indicate that CNS candidiasis in IC is common, is under-investigated, is difficult to diagnose, and is devastating in its impact on the neonate. These results emphasize the need for routine CSF analysis and other diagnostic modalities, including head imaging in infants under investigation for this infection, or after a positive sterile site or urine culture of *Candida*.

Invasive candidiasis occurred at a rate of 2.2% in ELBW infants and 1.5% in VLBW infants, with the highest rate of 4.2% in the lowest birth weight category (<750 g). Our rates are comparable to those reported in other studies in the pre-prophylaxis era with rates of 2 to 8% in ELBW and 1 to 2% in VLBW infants [4,16-18].

Necrotizing enterocolitis was the only independent risk factor for IC. Disruption of intestinal mucosal integrity in NEC appears to be a critical factor in facilitating the translocation of intra-intestinal *Candida* into the bloodstream in these infants.

The IDSA guideline on the management of neonatal IC currently recommends that in nurseries with high rates of IC, fluconazole prophylaxis should be considered as a preventive strategy in ELBW infants [19]. Given the devastatingly poor outcomes that we have described in infants with birth weights <1250 g, our data support this recommendation, and in the face of bowel perforation or NEC, should be considered even in infants >1250 g. At this time, however, of the centers included in our study, only one employs routine prophylaxis for ELBW infants. Several other centers use an individualized strategy based on risk factors.

One limitation of this study is that the unexpectedly high rate of congenital IC reduced the sample available for risk factor analysis. This limited our ability to examine more than 3 to 4 covariates simultaneously in the multivariable model and may have limited the number of factors that were identified as significant in the univariate analysis. In addition, the high mortality rate reduced the number of survivors remaining for neurodevelopmental assessment, especially those with CNS candidiasis. Data on timing of central line removal in relation to time of first positive blood culture was not available, and prevented analysis of the contribution of this factor to mortality. However, our follow-up rate of 97% (101/104) allowed us to confidently report neurodevelopmental outcome in survivors and correctly report the association of NDI with IC.

Although we have previously reported mortality outcomes in neonates with isolated *Candida* UTI (25% in ELBW, 30% in infants <1250 g and 27% in VLBW infants) [8], we were not able to address NDI in this group in our study as they did not receive long term follow-up. Based on the data in our previous study, including infants with isolated UTI would have resulted in similar mortality rates (42% in VLBW and 45% in ELBW).

This is the first prospective multicenter study to address risk factors and long-term outcome in neonatal candidiasis with a matched control group. Its strict inclusion criteria, high follow-up rate and multicenter design are significant strengths. We identified high mortality and high rates of neurodevelopmental impairment, including deafness, in cases compared to controls. We also identified high rates of CNS and congenital candidiasis and highlight the difficulties of diagnosing CNS infection with confidence. Future studies should be directed at exploring early diagnostic approaches for determining the presence of CNS candidiasis and at treatment interventions aimed at reducing unfavourable outcome in neonates with invasive candidiasis.

## Competing interests

All authors in this study declare that they have no financial or non-financial competing interests in relation to this manuscript.

## Authors’ contributions

MB analyzed and interpreted the data, drafted and revised the manuscript. KOB participated in acquisition and interpretation of the data, and drafting and revision of the manuscript. JLR participated in the conception and design of the study, acquisition of data, analysis and interpretation of the data, and drafting and revision of the manuscript. DHD participated in the conception and design of the study, acquisition of data, analysis and interpretation of the data, and drafting and revision of the manuscript. KS coordinated the study, recruited patients, oversaw the acquisition of data, and analyzed data. EA participated in the conception and design of the study, acquisition of data, analysis and interpretation of the data, and drafting and revision of the manuscript. JML participated in the conception and design of the study, acquisition of data, analysis and interpretation of the data, and drafting and revision of the manuscript. NLS participated in the acquisition of data and drafting and revision of the manuscript. RS participated in the acquisition of data and drafting and revision of the manuscript. AS participated in the acquisition of data and drafting and revision of the manuscript. BT participated in the conception and design of the study, acquisition of data and drafting and revision of the manuscript. LDR participated in the acquisition of data and drafting and revision of the manuscript. ER participated in the acquisition of data and drafting and revision of the manuscript. CH participated in the acquisition of data and drafting and revision of the manuscript. LK participated in the acquisition of data and drafting and revision of the manuscript. SER participated in the conception and design of the study, acquisition of data, analysis and interpretation of the data, and drafting and revision of the manuscript. All authors have given final approval of the version to be published and agree to be accountable for all aspects of the work.

## Pre-publication history

The pre-publication history for this paper can be accessed here:

http://www.biomedcentral.com/1471-2334/14/327/prepub
